# H3K27ac-activated EGFR-AS1 promotes cell growth in cervical cancer through ACTN4-mediated WNT pathway

**DOI:** 10.1186/s13062-021-00315-5

**Published:** 2022-01-08

**Authors:** Jingyan Li, Hongbing Wang

**Affiliations:** 1 Zibo Maternal and Child Health Hospital of Shandong Province, Zibo, 255000 Shandong China; 2 Department of Gynecology and Oncology, Hubei Cancer Hospital Affiliated to Tongji Medical College of Huazhong University of Science and Technology, Hongshan District, No. 116 Zhuodaoquan South Road, Wuhan, 430079 Hubei China

**Keywords:** H3K27 acetylation, EGFR-AS1, Cervical cancer, WNT pathway

## Abstract

**Background:**

Recently, extensive studies unveiled that lncRNAs exert critical function in the development and progression of cervical cancer (CC). EGFR-AS1 is a novel lncRNA which has not been well-explored in CC.

**Aims:**

Our study aimed to research the function and molecular mechanism of EGFR-AS1 in CC cells. qRT-PCR analysis was performed to detect gene expression. Colony formation, EdU, flow cytometry, TUNEL, western blot and transwell assays were performed to assess the effect of EGFR-AS1 on CC cell growth. The regulatory mechanism of EGFR-AS1 was dug out through mechanism experiments.

**Results:**

EGFR-AS1 was notably overexpressed in CC cell lines. Loss-of-functional experiments revealed that EGFR-AS1 promoted CC cell proliferation, migration and invasion, and suppressed cell apoptosis. Mechanistically, up-regulation of EGFR-AS1 was attributed to the activation of H3K27 acetylation (H3K27ac). Further, EGFR-AS1 was revealed to function as miR-2355-5p sponge. Additionally, miR-2355-5p was down-regulated in CC cells and ACTN4 was identified as a target gene of miR-2355-5p. Ultimately, overexpressed ACTN4 could reserve the suppressive role of EGFR-AS1 silencing in CC cell growth. Last but not least, EGFR-AS1 facilitated CC cell growth via ACTN4-mediated WNT pathway.

**Conclusions:**

H3K27ac-activated EGFR-AS1 sponged miR-2355-5p and promoted CC cell growth through ACTN4-mediated WNT pathway.

**Supplementary Information:**

The online version contains supplementary material available at 10.1186/s13062-021-00315-5.

## Background

As a common malignant gynecologic cancer, cervical cancer (CC) is considered as the major cause of mortality associated with cancers among female population [1]. Therapeutic approaches, such as surgery, radiotherapy and chemotherapy, have been improved in recent years; nevertheless, the long-term survival rate of CC patients is still unsatisfied with a high rate of recurrence, and metastatic CC is incurable [2]. Hence, it’s urgent to explore the potential molecular mechanisms underlying CC progression and develop the new therapeutic intervention for the patients who suffered from CC.

Recently, non-coding RNAs (ncRNAs) have been identified as novel biomarkers of diagnosis or underlying targets of therapy in diverse cancers [3]. Importantly, long non-coding RNAs (lncRNAs), a subtype of ncRNAs, was limited to encode proteins and longer than 200 nucleotides in length [4]. They have been reported to be expressed aberrantly and played the pivotal role in biological courses. Increasing studies manifested that lncRNA are frequently dysregulated in multiple cancer types, and a series of lncRNAs are related to cancer recurrence and unfavorable prognosis [5]. For example, lncRNA-PRLB has been uncovered as a novel lncRNA in breast cancer and expressed at a high level in cancer cells, and patients with high PRLB level present a shorter survival time [6]. LncRNA MT1JP has been identified as a tumor inhibitor in gastric cancer and suppresses cell proliferation and migration by targeting miR-214-3p/RUNX3 [7]. Another evidence suggests that LncRNA SNHG1 plays an oncogenic role in lung cancer and boosts cell proliferation and invasion via sponging miR-145-5p and up-regulating MTDH [8]. In recent years, research work regarding the relation between lncRNAs and CC has been accumulated [9, 10]. Importantly, biological role and underlying mechanisms of dysregulated lncRNAs has also unearthed in CC progression [11, 12]. However, it is still needed to make more efforts for seeking out the extra biomarkers for the patients with CC. LncRNA EGFR antisense RNA 1 (EGFR-AS1) has been proposed to work as a tumor facilitator in gastric cancer [13] and renal cancer [14], whereas its functional role and potential molecular mechanism have not been well explored in CC.

This research was performed to detect the expression level of EGFR-AS1 in CC cell and explored its functional effect on multiple biological behaviors of CC cells. Additionally, this study also tried to figure out the molecular mechanism of EGFR-AS1 in CC cells. These findings might provide a meaningful theoretical basis for exploring therapeutic methods for CC patients.

## Results

### EGFR-AS1 is highly expressed in CC cells and EGFR-AS1 knockdown hampers CC cell growth

**Fig. 1:**
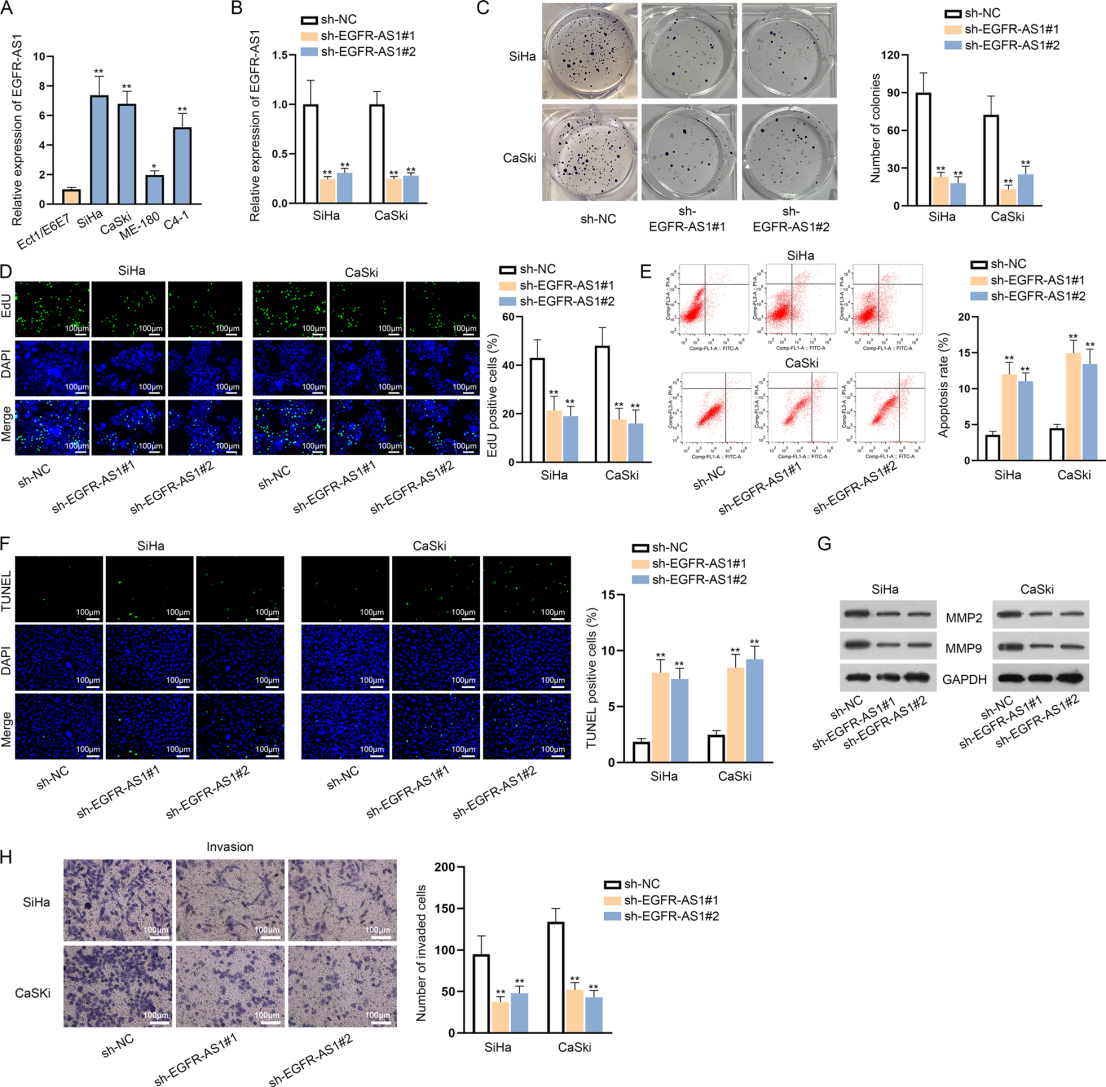
EGFR-AS1 is highly expressed in CC cells and EGFR-AS1 knockdown hampers CC cell growth. **a** EGFR-AS1 expression in CC cell lines and human normal cervical cell line was detected by qRT-PCR. **b** The transfection of sh-EGFR-AS1#1/2 was detected via qRT-PCR. **c**, **d** Colony formation and EdU assays were performed to evaluate the effect of EGFR-AS1 knockdown on CC cell proliferation. **e**, **f** The apoptosis in EGFR-AS1 silenced cells was tested via flow cytometry analysis and TUNEL assay. **g**, **h** Western blot assay and transwell assay were carried out for assessing cell migration and invasion. *P < 0.05; **P < 0.01. Scale bar 100 µm

To probe the expression pattern of EGFR-AS1 in CC, we detected its expression in CC cell lines (SiHa, CaSki, ME-180 and C4-1) and human normal cervical cell line (Ect1/E6E7) using qRT-PCR. The results indicated that EGFR-AS1 was overtly up-regulated in CC cell lines compared with Ect1/E6E7, especially in SiHa and CaSki cell lines (Fig. 1a). Later, we verified the functional role of EGFR-AS1 in CC via loss-of-function assays. Before the experiments, we stably silenced EGFR-AS1 expression in SiHa and CaSki cells by transfecting sh-EGFR-AS1#1/2 (Fig. 1b). Colony formation and EdU assays demonstrated that EGFR-AS1 silencing repressed the proliferation of SiHa and CaSki cells (Fig. 1c, d). Then, cell apoptosis was estimated in flow cytometry analysis and TUNEL assay. It was displayed that the apoptosis of SiHa and CaSki cells was stimulated upon EGFR-AS1 knockdown (Fig. 1e, f). Subsequently, the effect of EGFR-AS1 on CC cell migration and invasion was evaluated. Western blot assay elucidated that the levels of migration-related proteins (MMP2 and MMP9) were decreased by sh-EGFR-AS1 transfection in SiHa and CaSki cells (Fig. 1g). Through transwell assay, we observed that the knockdown of EGFR-AS1 suppressed the invasion of SiHa and CaSki cells (Fig. 1h). In brief, EGFR-AS1 is highly expressed in CC cells and EGFR-AS1 knockdown hampers CC cell growth.

### EGFR-AS1 is induced by CBP-mediated H3K27ac in CC cells

**Fig. 2:**
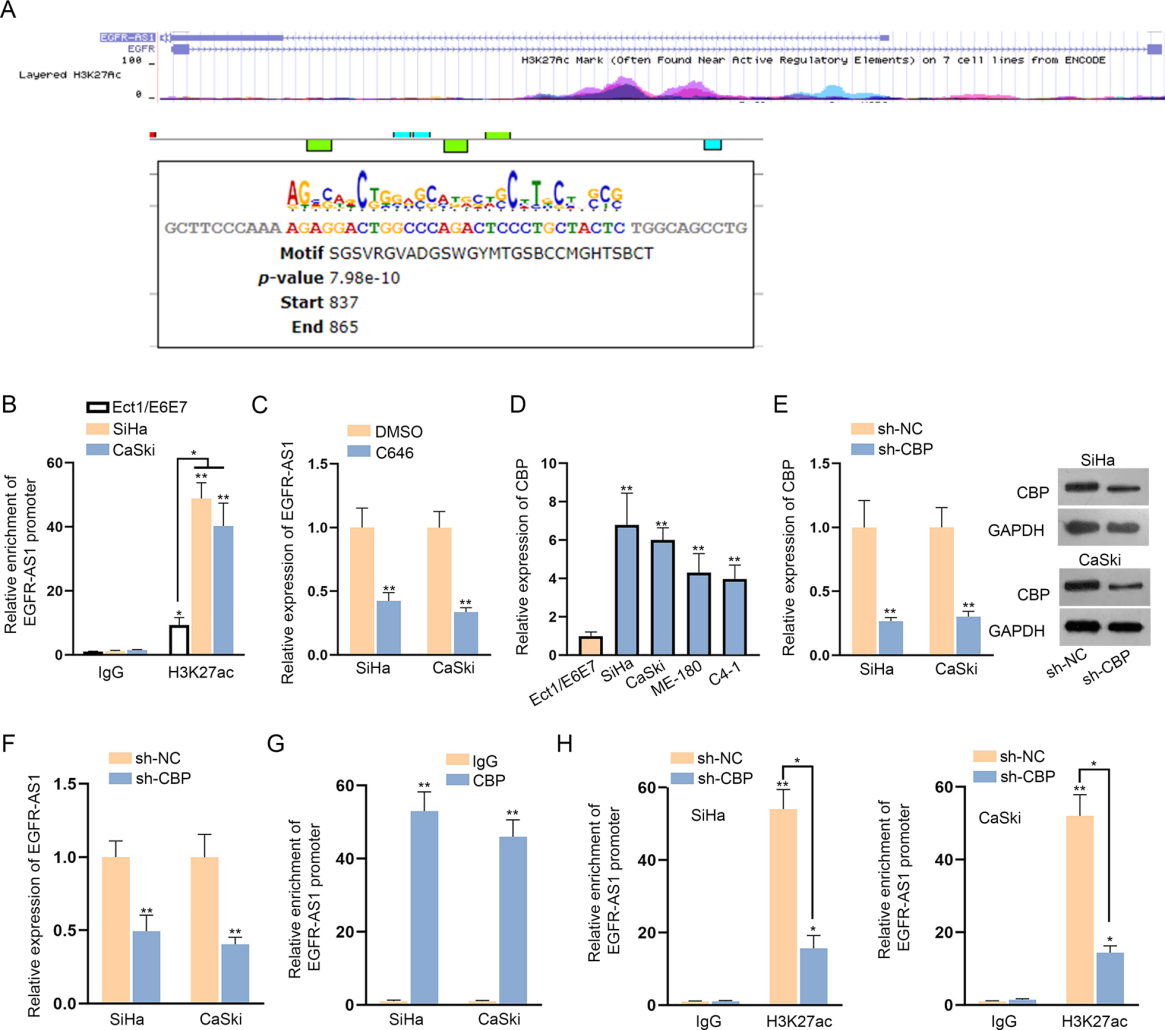
EGFR-AS1 is induced by CBP-mediated H3K27ac in CC cells. **a** The enrichment of H3K27ac at EGFR-AS1 promoter region and the amplified region were predicted by UCSC database. **b** H3K27ac level in SiHa, CaSki and Ect1/E6E7 cells at EGFR-AS1 promoter region was confirmed by ChIP assay. **c** EGFR-AS1 expression in CC cells transfected with C646 or DMSO was detected by qRT-PCR. **d** CBP expression in CC cell lines and human normal cervical cell line was detected by qRT-PCR. **e** CBP mRNA and protein levels were evaluated in sh-CBP transfected cells through qRT-PCR and western blot assays. **f** qRT-PCR was utilized for evaluating the effect of CBP depletion on EGFR-AS1 expression. **g** ChIP assay was performed to assess CBP enrichment at EGFR-AS1 promoter region. **h** The binding of H3K27ac to EGFR-AS1 promoter in sh-CBP group or sh-NC group was determined by ChIP assay. *P < 0.05; **P < 0.01

Later, we explored the mechanism of EGFR-AS1 up-regulation in CC cells. Existing studies implied that lncRNAs could be activated by H3K27ac at the transcriptional level [15, 16]. Using UCSC (http://genome.ucsc.edu/), high enrichment of H3K27ac was presented at EGFR-AS1 promoter region, and the amplified region in the following ChIP assay was also displayed (Fig. 2a). Therefore, we postulated that EGFR-AS1 up-regulation was caused by H3K27ac at its promoter region. To test this speculation, we tested the level of H3K27ac on EGFR-AS1 promoter in CC cell lines (SiHa and CaSki) and normal cervical cell line (Ect1/E6E7). It was found that SiHa and CaSki cell lines presented a higher level of H3K27ac than Ect1/E6E7 cell line (Fig. 2b). Later, C646, histone acetyltransferase (HAT) inhibitor, was utilized to treat CC cells, and we found that EGFR-AS1 expression was reduced in C646 treated cells in comparison of cells treated with DMSO (Fig. 2c). Above results pointed out that EGFR-AS1 was up-regulated because of H3K27ac. It is well known that CBP plays a critical role in histone acetylation and gene transcription [17]. Hence, we investigated whether CBP mediated the binding of H3K27ac at the promoter region of EGFR-AS1. High CBP expression in CC cell lines was affirmed by qRT-PCR (Fig. 2d). Then, we knocked down CBP mRNA and protein expressions in SiHa and CaSki cells (Fig. 2e). Further, we found that EGFR-AS1 expression was decreased in CBP silenced cells (Fig. 2f). In addition, results of ChIP assay demonstrated the notable enrichment of EGFR-AS1 promoter in CBP precipitates (Fig. 2g), indicating the binding of CBP to EGFR-AS1 promoter. Importantly, we confirmed that CBP silencing weakened the interaction between H3K27ac and EGFR-AS1 promoter (Fig. 2h). Taken all together, EGFR-AS1 is induced by CBP-mediated H3K27ac in CC cells.

### EGFR-AS1 functions as miR-2355-5p sponge

**Fig. 3:**
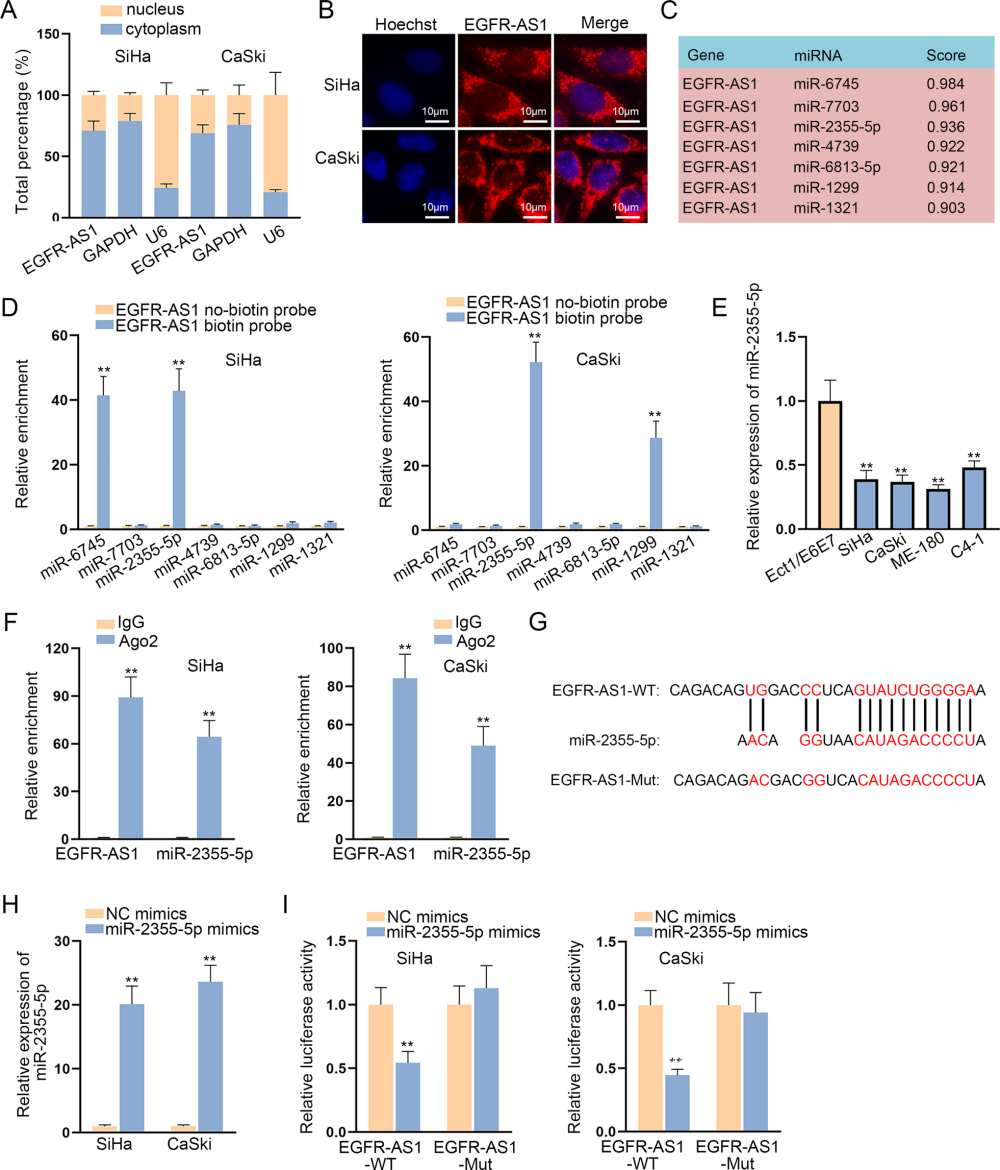
EGFR-AS1 functions as miR-2355-5p sponge. **a**, **b** Subcellular fractionation and FISH assays were conducted for detecting the distribution of EGFR-AS1 in CC cells (Scale bar: 10 µm). **c** DIANA tool was applied to predict candidate miRNAs that could bind to EGFR-AS1. **d** RNA pull down assay was employed to screen out the potential miRNAs for EGFR-AS1 in CC cells. **e** MiR-2355-5p expression was measured in CC cell lines and human normal cervical cell line by qRT-PCR. **f** RIP assay was used to identify the enrichment of EGFR-AS1 and miR-2355-5p in RNA-induced silencing complex (RISC). **g** The binding sites between EGFR-AS1 (wild-type or mutant) and miR-2355-5p were displayed. **h** CC cells were transfected with miR-2355-5p mimics and the transfection efficiency was identified by qRT-PCR. **i** Luciferase activity of EGFR-AS1-WT or EGFR-AS1-MUT was evaluated in CC cells with the transfection of miR-2355-5p mimics or NC mimics. **P < 0.01

LncRNAs are widely reported to exhibit regulatory functions via participating in competing endogenous RNA (ceRNA) network in the cytoplasm of cancer cells [18, 19]. To confirm EGFR-AS1 localization in CC cells, we conducted subcellular fractionation and FISH experiments. The result disclosed the chief localization of EGFR-AS1 in the cytoplasm (Fig. 3a, b). Using DIANA (http://carolina.imis.athenainnovation.gr/diana-tools/web/index.php?r=lncbasev2/index), 7 potential miRNAs (score > 0.9) were obtained for EGFR-AS1 (Fig. 3c). RNA pull down assay confirmed only miR-2355-5p could potentially interact with EGFR-AS1 in SiHa and CaSki cell lines (Fig. 3d). In addition, miR-2355-5p showed a low expression level in CC cells (Fig. 3e). RIP assay manifested that both EGFR-AS1 and miR-2355-5p were remarkably precipitated by Ago2 antibody (Fig. 3f). Then, the binding sequence of miR-2355-5p on EGFR-AS1 was predicted and mutant binding site was constructed (Fig. 3g). As shown, miR-2355-5p expression was elevated in SiHa and CaSki cells with the transfection of miR-2355-5p mimics (Fig. 3h). From luciferase reporter assay, we observed that miR-2355-5p up-regulation merely lessened the luciferase activity of EGFR-AS1-WT (Fig. 3i). To sum up, EGFR-AS1 functions as miR-2355-5p sponge.

### ACTN4 is identified as the target of miR-2355-5p

**Fig. 4:**
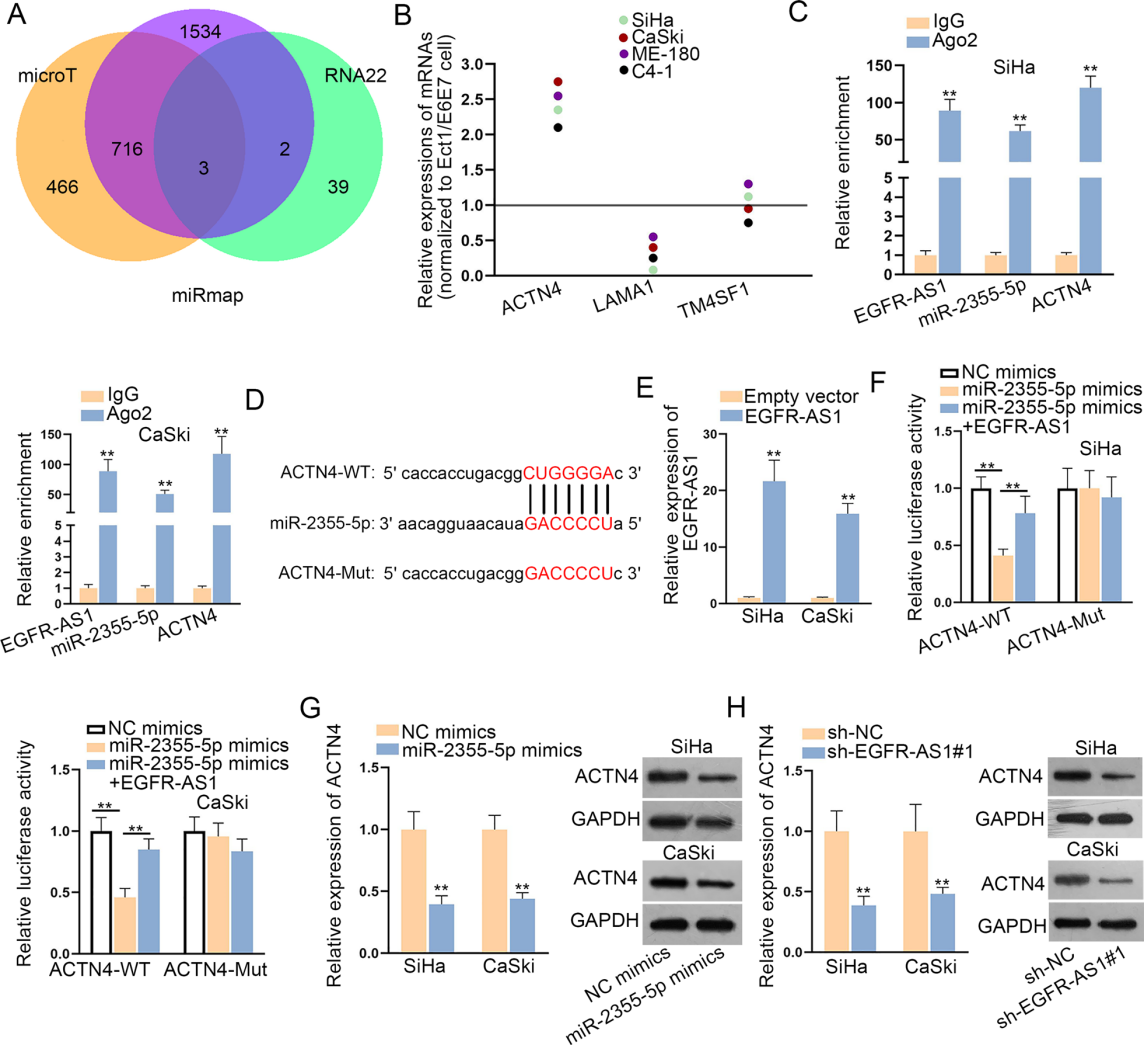
ACTN4 is identified as the target of miR-2355-5p. **a** Three mRNAs with potential binding to miR-2355-5p were predicted by microT, miRmap, and RNA22 databases. **b** Expression levels of predicted mRNAs in CC cell lines and human normal cervical cell line was detected via qRT-PCR. **c** The enrichment of EGFR-AS1, miR-2355-5p and ACTN4 in RISC was certified by RIP assay. **d** The binding sites between ACTN4 (wild-type or mutant) and miR-2355-5p was manifested. **e** EGFR-AS1 expression was assessed after transfecting overexpression plasmid (labeled as EGFR-AS1) by qRT-PCR. **f** Luciferase activity of ACTN4-WT or ACTN4-MUT was estimated in CC cells transfected the indicated plasmids. **g**, **h** ACTN4 mRNA and protein levels in miR-2355-5p mimics or sh-EGFR-AS1#1 transfected cells were affirmed by qRT-PCR and western blot assays. **P < 0.01

Next, we sought the target gene of miR-2355-5p in the regulation mechanism. Using prediction databases (microT, miRmap, and RNA22), 3 targets of miR-2355-5p were found (Fig. 4a). Through qRT-PCR analysis, we observed that expression of ACTN4 was lifted in CC cells while LAMA1 was down-regulated and TM4SF1 didn’t show expression difference (Fig. 4b). RIP assay further revealed the co-existence of EGFR-AS1, miR-2355-5p and ACTN4 in Ago2 group (Fig. 4c). As Fig. 4d presented, wild-type/mutant binding sites of ACTN4 for miR-2355-5p were predicted. Then, EGFR-AS1 expression was up-regulated for the follow-up experiments by transfecting overexpression plasmid (Fig. 4e). Through luciferase reporter assay, we viewed that EGFR-AS1 overexpression recovered the reduced ACTN4-WT luciferase activity in miR-2355-5p up-regulated cells while that of ACTN4-MUT was not affected (Fig. 4f). Subsequently, it was delineated that ACTN4 mRNA and protein levels were alleviated by transfecting miR-2355-5p mimics or sh-EGFR-AS1#1 via qRT-PCR and western blot (Fig. 4g, h). To be concluded, ACTN4 is identified as the target of miR-2355-5p.

### EGFR-AS1 enhances CC cell growth via up-regulating ACTN4

**Fig. 5:**
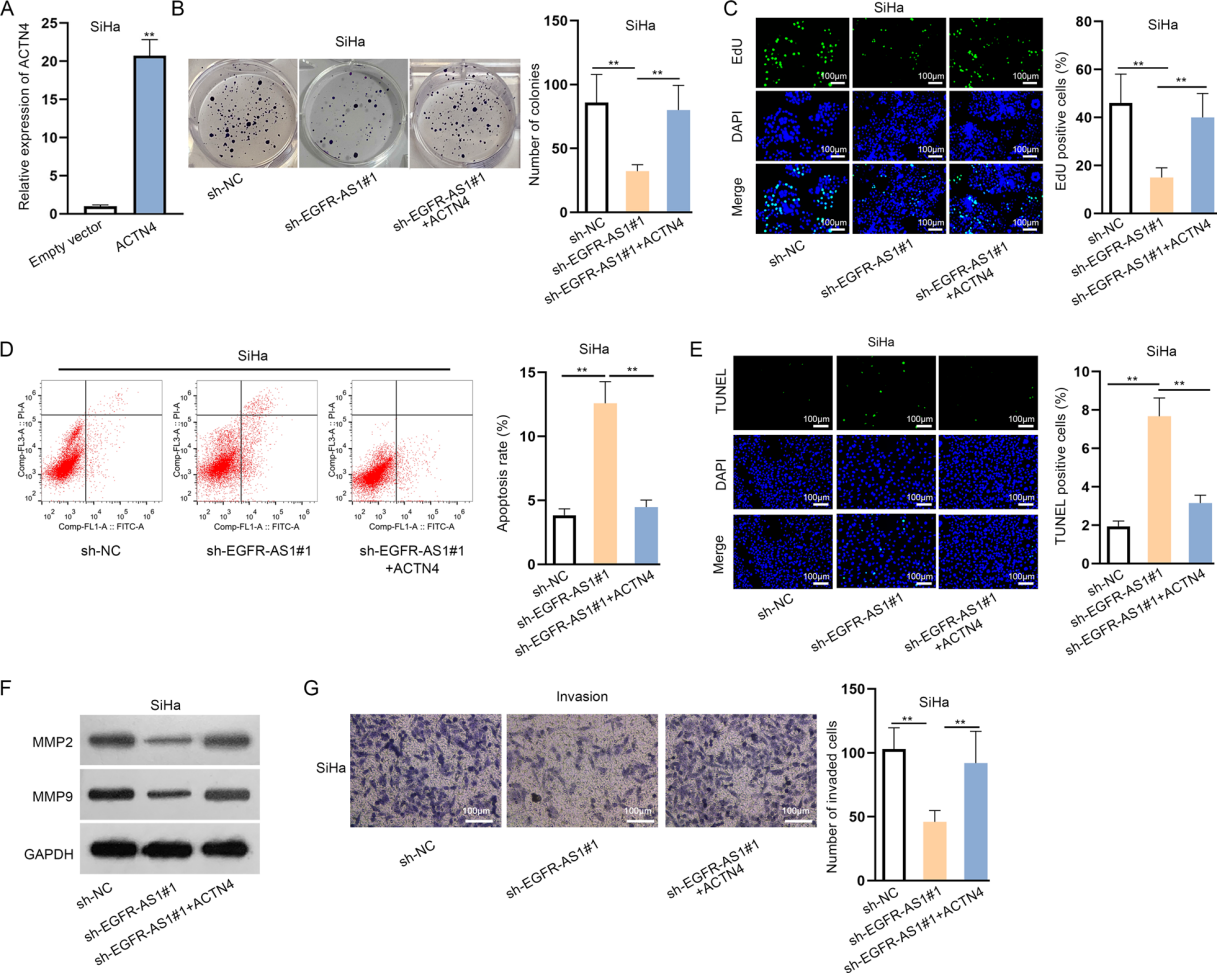
EGFR-AS1 enhances CC cell growth via up-regulating ACTN4. **a** Transfection efficiency of pcDNA3.1/ACTN4 (labeled as ACTN4) in SiHa cells were examined by qRT-PCR. **b**, **c** The proliferation was analyzed by colony formation and EdU assays in SiHa cells transfected with sh-NC, sh-EGFR-AS1#1 and sh-EGFR-AS1#1 + ACTN4. **d**, **e** The effect of sh-NC, sh-EGFR-AS1#1 and sh-EGFR-AS1#1 + ACTN4 on cell apoptosis was evaluated by flow cytometry analysis and TUNEL assay. **f**, **g** Cell migration and invasion were estimated through western blot and transwell assays in SiHa cells with the transfection of sh-NC, sh-EGFR-AS1#1 and sh-EGFR-AS1#1 + ACTN4. **P < 0.01. Scale bar: 100 µm

To verify the role of EGFR-AS1/ACTN4 axis in CC cell growth, we carried out functional experiments in a rescue manner. Firstly, ACTN4 expression was augmented in SiHa cells (Fig. 5a). Based on colony formation and EdU assays, the suppressed proliferation was noticed in EGFR-AS1 silenced cells, whereas ACTN4 overexpression abolished this effect (Fig. 5b, c). Via flow cytometry analysis and TUNEL assay, the induced apoptosis by sh-EGFR-AS1#1 was impaired by overexpressed ACTN4 (Fig. 5d, e). Western blot assay uncovered that ACTN4 up-regulation abrogated the decreased migration-related protein levels in EGFR-AS1 down-regulated cells (Fig. 5f). Moreover, the suppressive effect of EGFR-AS1 silencing on cell invasion was found to be neutralized by up-regulated ACTN4 in transwell assay (Fig. 5g). Namely, EGFR-AS1 enhances CC cell growth via up-regulating ACTN4.

### EGFR-AS1 activates ACTN4-mediated WNT pathway in CC cells

**Fig. 6:**
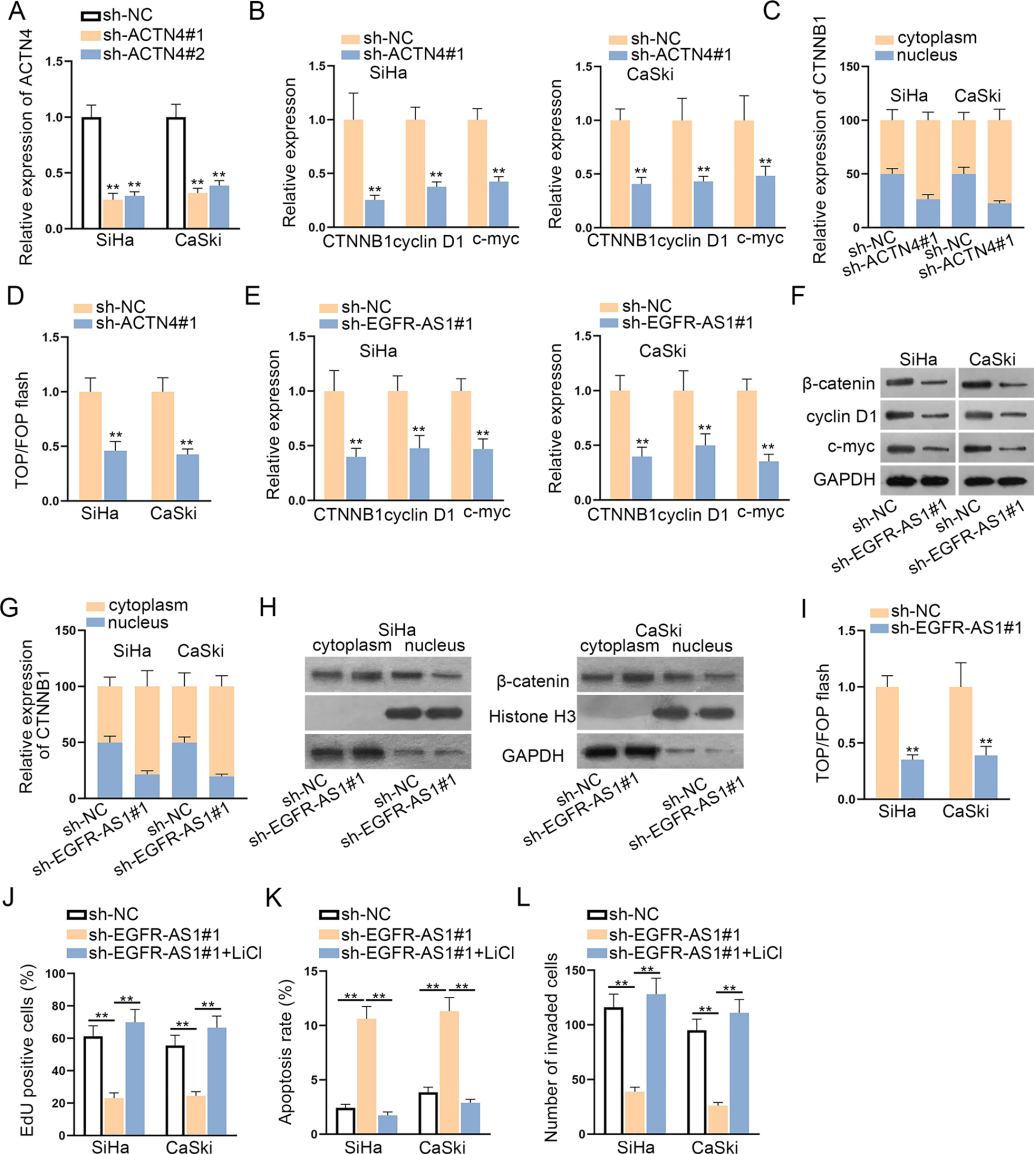
EGFR-AS1 activates ACTN4-mediated WNT pathway in CC cells. **a** The knockdown efficacy of sh-ACTN4#1/2 on was tested by qRT-PCR in CC cells. **b** Expression levels of WNT pathway downstream genes in sh-ACTN4#1 transfected cells were detected by qRT-PCR in CC cells. **c** The effect of ACTN4 deficiency on the nuclear translocation of CTNNB1 (β-catenin) was detected by subcellular fractionation assay. **d** TOP/FOP flash assay was performed to measure the activity of WNT pathway upon ACTN4 knockdown. **e**, **f** Detection of mRNA and protein levels of WNT pathway downstream genes in CC cells transfected with sh-EGFR-AS1#1 was detected by qRT-PCR and western blot assays. **g**, **h** The nuclear translocation of CTNNB1 (β-catenin) was observed in sh-EGFR-AS1#1 group and sh-NC group. **i** Impact of EGFR-AS1 silencing on WNT pathway activity was detected. **j**–**l** Cell proliferation, apoptosis and invasion in cell transfected with sh-NC, sh-EGFR-AS1#1 and sh-EGFR-AS1#1 + LiCl were assessed by EdU, flow cytometry and transwell assays. **P < 0.01

ACTN4 was previously reported to enhance CC cell proliferation by promoting WNT pathway activation [20]. Here, we silenced ACTN4 expression in CC cells (Fig. 6a), and found that the downstream gene expressions (CTNNB1, cyclin D1 and c-myc) of WNT pathway declined (Fig. 6b). Besides, the nuclear translocation of CTNNB1 (β-catenin) was also lessened upon ACTN4 knockdown (Fig. 6c). TOP/FOP flash assay unveiled the repressive role of sh-ACTN4#1 in WNT pathway activity (Fig. 6d). Above data suggested that ACTN4 affected WNT pathway in CC cells. In this study, ACTN4 was found to involve in EGFR-AS1-mediated ceRNA network. Thus, the regulatory role of EGFR-AS1 in WNT pathway was further analyzed. Firstly, the levels of WNT pathway downstream genes were detected in sh-EGFR-AS1#1 transfected cells. As a result, the mRNA and protein levels of CTNNB1 (β-catenin), cyclin D1 and c-myc were attenuated by sh-EGFR-AS1#1 transfection (Fig. 6e, f). In addition, EGFR-AS1 depletion refrained the nuclear translocation of CTNNB1 (β-catenin) of CC cells (Fig. 6g, h). Furthermore, the activity of WNT pathway was impaired by EGFR-AS1 silencing (Fig. 6i). For further study, we used WNT pathway activator (LiCl) to carry out rescue experiments. As we observed, the treatment of LiCl recovered the effect of sh-EGFR-AS1#1 on CC cell proliferation, apoptosis and invasion (Fig. 6j–l). Based on recent literature, CHIR99021 is known as GSK-3β inhibitor and WNT activator [21, 22]. Hence, we utilized CHIR99021 to do a series of rescue assays. As shown in Additional file 1: Fig. S1A–C, EGFR-AS1 depletion restricted proliferation and invasion of CC cells, while enhancing the cell apoptosis. However, with the treatment of CHIR99021, these effects were greatly abrogated. Collectively, EGFR-AS1 activates ACTN4-mediated WNT pathway in CC.

## Discussion

Accumulating studies have expounded that lncRNAs with abnormal expressions serve essential parts in human cancers, including CC. For example, lncRNA LINC00675 is up-regulated in CC cells and fortifies cell proliferation and invasion via impacting WNT pathway in CC [23]. LncRNA HOTAIR regulates radiotherapy resistance, epithelial-mesenchymal transition and autophagy in CC through WNT pathway [24]. LncRNA DANCR is associated with poor prognosis and promotes CC cell growth by targeting miR-335-5p/ROCK1 axis [25]. In consequence, it is indispensable to search extra lncRNAs and understand their underlying mechanisms for the exploration of therapeutic strategies. The study on biological function and molecular mechanisms of EGFR-AS1 has been researched in gastric cancer [13] and renal cancer [14]. However, the research about those of EGFR-AS1 in CC remains largely unclear. Here, we found the high expression level of EGFR-AS1 in CC cells. Loss-of-function investigations disclosed that EGFR-AS1 silencing impaired CC cell proliferation, migration and invasion and ascended cell apoptosis, which exhibited the oncogenic property of EGFR-AS1 in CC.

H3K27ac is known as a common type of histone modification [26], which correlates with the active enhancer modulatory elements to activate gene expression [27]. In recent reports, H3K27ac at promoter regions leads to the up-regulation of certain lncRNAs and thereby regulates tumor progression [15, 16]. Additionally, CBP was considered as a pivotal regulator of the histone acetylation [17]. Herein, we found that H3K27ac was highly enriched in EGFR-AS1 promoter region. Besides, EGFR-AS1 expression was reduced by HAT inhibitor (C646), suggesting that EGFR-AS1 up-regulation was caused by H3K27ac modification. Accordingly, we found that CBP interacted with EGFR-AS1 promoter to trigger the H3K27ac and induce the up-regulation of EGFR-AS1.

Subsequently, we probed the molecular mechanism underlying EGFR-AS1. Previously, mounting reports have confirmed that lncRNAs act as ceRNAs to alleviate the modulatory impact of microRNAs (miRNAs) on target mRNAs at post-transcriptional level [28]. MiRNAs are single-stranded non-coding RNAs with 22–24 nucleotides in length and modulate a wide range of cellular processes [29]. For example, miRNA-135b induces cell apoptosis and inhibits cisplatin resistance and proliferation by regulating MAPK in gastric cancer [30]. MiR-4516 functions as a novel oncogene and predicts poor prognosis by targeting PTPN14 in human glioblastoma [31]. MiR-2355-5p is a novel miRNA which has neither been studied in CC nor in other cancers. In this study, EGFR-AS1 was determined as a cytoplasmic RNA and functioned as a sponge of miR-2355-5p in CC cells.

ACTN4 has been reported as a tumor promoter in gastric cancer [32], colorectal cancer [33] and pancreatic cancer [34]. Importantly, ACTN4 has been reported to activate WNT pathway in CC [35]. In this study, ACTN4 was identified as a target gene of miR-2355-5p, and ACTN4 overexpression restored EGFR-AS1 silencing-mediated suppression on CC cell growth. Furthermore, EGFR-AS1 activated ACTN4-induced WNT pathway in CC cells.

## Conclusions

In conclusion, this study explored the regulatory mechanism of EGFR-AS1 in CC cells, and revealed that H3K27ac-activated EGFR-AS1 promoted CC cell growth through ACTN4-mediated WNT pathway, suggesting the meaningful revelation for investigating the therapeutic methods for patients who are diagnosed with CC.

## Methods

### Cell culture and treatment

Human CC cell lines (SiHa, CaSki, ME-180, C4-1) and human normal cervical cell line (Ect1/E6E7), from ATCC (Rockville, Maryland), were allowed to grow under 37 °C and 5% CO_2_ in the DMEM (Invitrogen, Carlsbad, CA). The 1% antibiotics and 10% FBS, both from Invitrogen, were acquired for purpose of cell culture. Besides, 20 mmol/l of LiCl, 10 mM of DMSO and 20 nM of C646 were all purchased from Sigma Aldrich (St. Louis, MI) to treat SiHa and CaSki cells.

### qRT-PCR analysis

The total RNA from cultured cells were extracted with Invitrogen TRIzol reagent, then 1 μg of total RNA was prepared to synthesize cDNA. Expression levels of target genes were monitored through qRT-PCR with SYBR R Premix Ex TaqTM II (Takara, Shiga, Japan), processed by the 2^−ΔΔCt^ method and normalized to U6 or GAPDH.

### Cell transfection

The designed shRNAs were produced by Genepharma (Shanghai, China) to silence EGFR-AS1, CBP and ACTN4 employing the transfection kit Lipofectamine 2000 (Invitrogen). Negative control (sh-NC) was also obtained. The pcDNA3.1/EGFR-AS1, pcDNA3.1/ACTN4 and empty pcDNA3.1 vectors, along with miR-2355-5p mimics and NC mimics, were all acquired from Genepharma for 48 h of transfection.

### Colony formation

Cells in six-well plates (1000 cells/well) were cultivated for 14 days. Next, generated colonies were fixed with 4% paraformaldehyde and stained by 0.1% crystal violet. After washing in PBS, clones were counted.

### EdU assay

Cells in 24-well plates were transfected with designed plasmids and plated on the sterile coverslips. EdU assay kit (Ribobio, Guangzhou, China) was used in light of direction. Cell nucleus was double stained by EdU and DAPI, and then subjected to fluorescence microscopy (Olympus, Tokyo, Japan).

### Flow cytometry analysis

Annexin V-FITC/PI Apoptosis kit was acquired from Invitrogen for measuring apoptotic cells. 2 × 10^5^ cells were collected and added into the binding buffer. 15 min later, samples were assayed using FACSCalibur flow cytometer (BD Biosciences, San Jose, CA).

### TUNEL assay

Cultured CC cell apoptosis was also measured by TUNEL assay. Cells in 6-well plates were cultured on coverslips and fixed by 4% paraformaldehyde. In situ cell death detection kit (Minneapolis, MN) was used as per manual. Apoptotic CC cells were observed under fluorescence microscopy.

### Western blot

Total protein samples were separated with 10% SDS-PAGE and shifted to PVDF membranes. Following blocking in 5% nonfat milk, membranes were probed with primary antibodies (1:2000; Abcam, Cambridge, MA) and appropriate HRP-tagged secondary antibodies (1:5000; Abcam). After culturing in TBST solution, samples were quantified by ECL Prime Western Blotting Detection reagent (GE Healthcare, Chicago, IL).

### Transwell invasion analysis

Invasion assay was implemented using the 8-mm pore size Transwell chambers coated with matrigel (Corning, Corning, NY). Lower chamber was filled with complete medium; cells in serum-free medium were added to upper chamber. After 24 h, fixed cells were treated with 0.1% crystal violet dye and observed under light microscope.

### ChIP assay

Primer sequences of EGFR-AS1 promoter were specifically designed as followed. Forward: GATGGTGGGTGGAAAGGGAG (Start: 778); Reverse: CCTTTCGAATGGGCAGGAGT (Start: 1022). With ChIP kit (Millipore, Billerica, MA), ChIP assay in CC cells were conducted as designed. After the DNA and protein underwent cross-linking, the chromatin was fragmented by ultrasonic and immunoprecipitated with the specific antibody to H3K27ac or CBP. IgG antibody acted as control. qRT-PCR was used for detecting the relative DNA enrichment.

### Subcellular fractionation

With PARIS™ Kit, the cytoplasmic and nuclear fractions were severally isolated from processed CC cells in line with user guide (Ambion, Austin, TX). The isolated RNAs were assayed by qRT-PCR.

### FISH

RNA FISH assay was implemented using the EGFR-AS1-FISH probe synthesized by Ribobio as instructed by supplier. After staining nuclei with Hoechst, fluorescence microscopy was employed.

### RNA pull down assay

The protein extracts from cultured CC cells were mixed with the EGFR-AS1 biotin probe or EGFR-AS1 no-biotin probe, and then incubated with magnetic beads for 1 h. RNA enrichment in pull-downs was finally monitored.

### RIP assay

Cell extracts from CC cells were acquired and cultivated with the magnetic beads conjugated to specific antibodies including human Ago2 and normal control IgG. Immunoprecipitated RNAs were purified for qRT-PCR analysis.

### Luciferase reporter assay

The wild-type and mutated EGFR-AS1 or ACTN4 fragments covering miR-2355-5p binding sites were obtained and inserted to the pmirGLO reporter vector (Promega Corporation, Madison, WI). The formed EGFR-AS1-WT/Mut and ACTN4-WT/Mut reporter vectors were co-transfected to SiHa and CaSki cells with indicated transfection plasmids. 48 h later, luciferase reporter assay system (Promega) was applied. Besides, TOP/FOP-flash reporter vectors were available to assay the Wnt/β-catenin signaling activity as guided by manufacturer (Addgene, Cambridge, MA).

### Statistical analyses

Averaged results of more than 3 independent experiments were used, and data were all exhibited as SD. Prism 5.0 software (GraphPad Software, Inc., La Jolla, CA) was employed to analyze data by t test, one-way or two-way ANOVA, with p value below 0.05 as the threshold.

## Supplementary Information

**Additional file 1: Fig. S1**. SiHa and CaSki cells were subjected to different treatments: sh-NC, sh-EGFR-AS1#1 and sh-EGFR-AS1#1 + CHIR99021. (A–C) EdU, flow cytometry and transwell assays were performed to evaluate the proliferation, apoptosis and invasion of the indicated CC cells. **P < 0.01.

## Data Availability

Not applicable.

## Abbreviations

CC: Cervical cancer
ncRNAs: Non-coding RNAs
lncRNAs: Long noncoding RNAs
EGFR-AS1: EGFR antisense RNA 1
DMEM: Dulbecco’s Modified Eagle's Medium
FBS: Fetal bovine serum
DMSO: Dimethyl sulfoxide
qRT-PCR: Quantitative real-time polymerase chain reaction
GAPDH: Glyceraldehyde-3-phosphate dehydrogenase
shRNA: Short hairpin RNA
NC: Negative control
PBS: Phosphate buffer saline
PVDF: Polyvinylidene fluoride
ChIP: Chromatin immunoprecipitation
IgG: Immunoglobulin G
RIP: RNA immunoprecipitation
SD: Standard deviation
HAT: Histone acetyltransferase
FISH: Fluorescent in situ hybridization
ceRNA: Competing endogenous RNA
miRNAs: MicroRNAs
ACTN4: Alpha-actinin-4
RISC: RNA-induced silencing complex
