# Supplementary figures and images for: H3K27ac-activated EGFR-AS1 promotes cell growth in cervical cancer through ACTN4-mediated WNT pathway
Source: Biol Direct. 2022 Jan 8;17:3. doi: 10.1186/s13062-021-00315-5 (PMC8742952; doi:10.1186/s13062-021-00315-5)

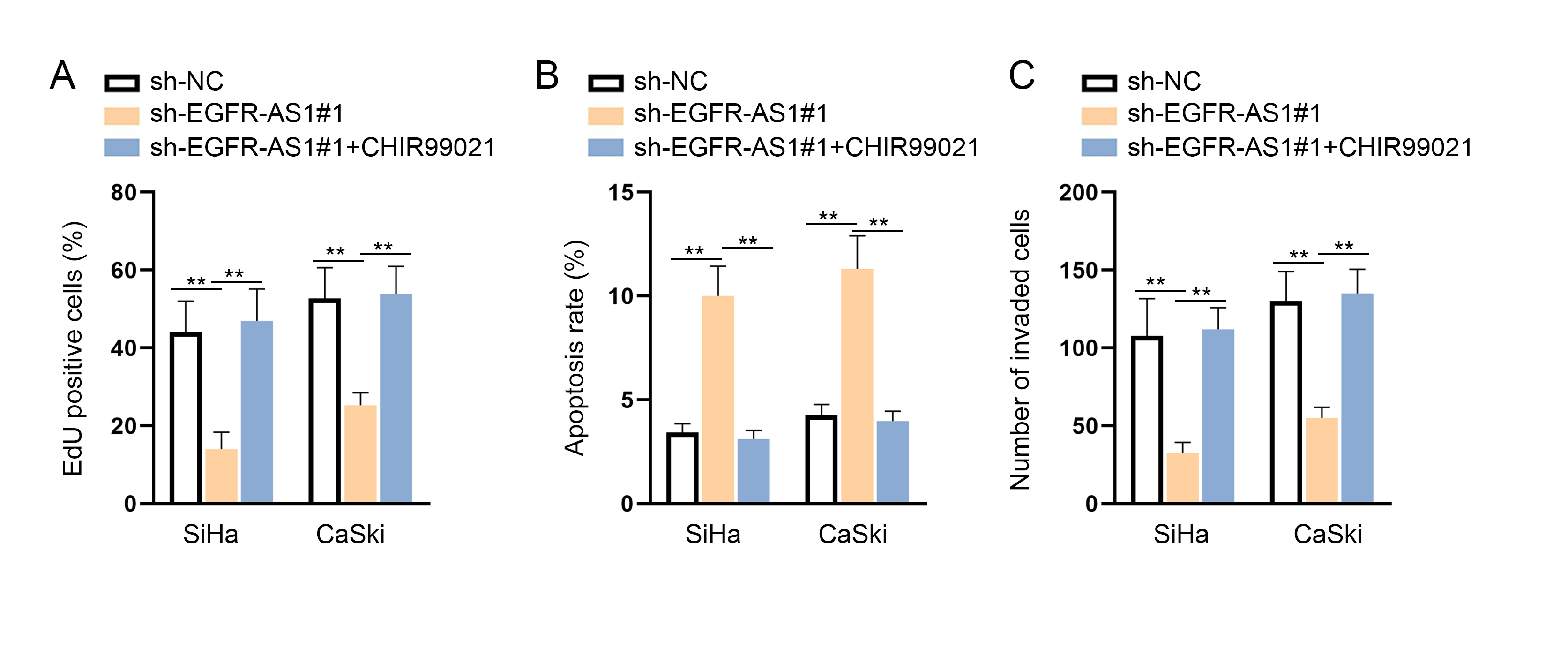

Supplement: Supplementary file 1 — Additional file 1: Fig. S1. SiHa and CaSki cells were subjected to different treatments: sh-NC, sh-EGFR-AS1#1 and sh-EGFR-AS1#1 + CHIR99021. (A–C) EdU, flow cytometry and transwell assays were performed to evaluate the proliferation, apoptosis and invasion of the indicated CC cells. **P < 0.01. [file 13062_2021_315_MOESM1_ESM.tif]
